# Why We Forget: Memory Encoding in the Aging Brain

**DOI:** 10.1002/alz.094679

**Published:** 2025-01-09

**Authors:** Lori L Beason‐Held, Andrea T Shafer, Nicole Calcagno, Jasmine Cooper, Melissa Kitner‐Triolo, Susan M. Resnick

**Affiliations:** ^1^ Laboratory of Behavioral Neuroscience, National Institute on Aging, NIH, Baltimore, MD USA; ^2^ Laboratory of Behavioral Neuroscience, National Institute on Aging, Intramural Research Program, Baltimore, MD USA

## Abstract

**Background:**

To further understand the neural basis of memory failure in the older brain, we examined the medial temporal lobe (MTL) memory system in 297 cognitively normal participants (mean age = 74.46 (4.95 SD)) from the Baltimore Longitudinal Study of Aging (BLSA).

**Methods:**

Participants performed a face‐scene associative memory encoding task, where they saw a series of scenes with a face at the bottom during fMRI scanning. To help them remember, they were asked whether they thought the face was a good match for the place in the background. An hour after scanning, they were asked to indicate which of two faces was shown before with each place. Brain activation and functional connectivity patterns were examined during encoding trials they later remembered, trials they later forgot, and for encoding success (remembered–forgotten trials).

**Results:**

Brain activation patterns involved regions along the occipitotemporal visual pathway and included the hippocampus (HC), entorhinal cortex (ENT), and parahippocampal gyrus (PHG) for both remembered and forgotten stimuli. In contrast, connectivity differed between remembered and forgotten trials. Remembered encoding was associated with greater connectivity between HC, ENT, and PHG seed regions, and most cortical regions along the occipitotemporal visual pathway. This connectivity pattern was absent for forgotten items. Participant good/bad match perception of the face‐scene pairs during encoding showed bad matches were more frequently forgotten. Task reaction times were also slower for bad matches.

**Conclusions:**

Taken together, this suggests that forgetting may be linked to disruption of the medial temporal lobe networks of the brain, and to whether the features of a visual stimulus are perceived as discordant or mismatched.

This work was supported by the Intramural Research Program of the NIH, National Institute on Aging.

